# Diagnostic Utility of Superb Microvascular Imaging and Power Doppler Ultrasonography for Visualizing Enriched Microvascular Flow in Patients With Carpal Tunnel Syndrome

**DOI:** 10.3389/fneur.2022.832569

**Published:** 2022-03-31

**Authors:** Takeshi Endo, Yuichiro Matsui, Daisuke Kawamura, Atsushi Urita, Daisuke Momma, Mitsutoshi Ota, Hiroki Shibayama, Takahito Iwai, Mutsumi Nishida, Norimasa Iwasaki

**Affiliations:** ^1^Department of Orthopaedic Surgery, Faculty of Medicine and Graduate School of Medicine, Hokkaido University, Sapporo, Japan; ^2^Faculty of Dental Medicine, Hokkaido University, Sapporo, Japan; ^3^Center for Sports Medicine, Hokkaido University Hospital, Sapporo, Japan; ^4^Division of Laboratory and Transfusion Medicine, Diagnostic Center for Sonography, Hokkaido University Hospital, Sapporo, Japan

**Keywords:** median nerve, compression neuropathy, carpal tunnel syndrome, ultrasound imaging, superb microvascular image

## Abstract

Recent studies suggest that blood flow changes in the median nerve may help confirm a diagnosis of carpal tunnel syndrome (CTS). Herein, we examined the utility of superb microvascular imaging (SMI), a new ultrasonographic (US) technique for visualizing microvascular flow, for detecting blood flow differences between CTS patients and healthy controls. We performed a retrospective analysis of 28 hands with suspected CTS. Patients received both nerve conduction and US examinations. Ten healthy volunteers were enrolled as the control group. The nerve compression ratio and the blood flow signal area were quantified using color Doppler US (CDUS), power Doppler US (PDUS), and SMI. Correlation analyses between the blood flow signal area, the compound muscle action potential of the thenar muscle, and the nerve compression ratio were performed. As a result, the mean nerve compression ratio was found to be significantly higher in the CTS group. There were no differences in the blood flow signal area between the groups using CDUS, while PDUS and SMI showed higher blood flow signals in the CTS group. The blood flow signal area measured by SMI had stronger correlations with the compound muscle action potential amplitude and the nerve compression ratio than those for PDUS. The diagnostic utility of SMI was equivalent to PDUS, but superior to conventional CDUS. Nevertheless, the blood flow signal by SMI was more strongly correlated with the electrophysiological severity and compression ratio than for PDUS. Use of SMI in future studies may help clarify the underlying mechanisms of blood flow changes in CTS.

## Introduction

Carpal tunnel syndrome (CTS) is a common compressive neuropathy of the median nerve, especially in postmenopausal women (1, 2). Patients report numbness, pain, and weakness in the hand and digits innervated by the median nerve (1). The classical triad for diagnosis includes hypoesthesia restricted to the median nerve distribution, a positive Tinel sign, and a positive Phalen test (1). Recent studies have also reported the diagnostic utility of Carpal Tunnel Syndrome 6 (CTS-6), which includes nocturnal numbness, thenar atrophy and/or weakness, and loss of 2-point discrimination in addition to the classical triad (3, 4). However, these signs may be unspecific for CTS and insufficient to differentiate from other disorders such as polyneuropathy or cervical radiculopathy (2). A nerve conduction study (NCS) is an established supplemental diagnostic tool used to assess neuropathy severity (5) and support surgical indication (6). However, NCS can only assess dysfunction of large myelinated fibers, but not small myelinated and non-myelinated fibers (2).

Recent studies have reported the utility of ultrasound (US) examination for CTS diagnosis (7, 8). The major focus of those studies involved the morphological changes of the median nerve, with the cross-sectional area (CSA) of the nerve calculated at the inlet of the carpal tunnel. However, the optimal cutoff value of the CSA remains controversial (8). Recently, US blood flow analysis has been examined as an alternative diagnostic technique for CTS (9–17). For example, increased blood flow in the median nerve of CTS patients was reported using contrast-enhanced US (17). However, this technique is not widely used because it is expensive and invasive. Superb microvascular imaging (SMI) is a new vascular imaging technique. It enables separation of low velocity blood flow signals from overlaying tissue motion artifacts instead of suppressing these low flow signals and high-resolution visualization of low velocity blood flow, which cannot be achieved in color Doppler US (CDUS) or power Doppler US (PDUS). SMI performed with the Aplio 500 device was also reported to be superior to CDUS and PDUS for visualizing intraneural blood flow (18, 19). Nevertheless, the utility of SMI for detecting CTS remains controversial. Furthermore, to our knowledge there are currently no reports of SMI using the latest Aplio device series.

In the present study, we examined the utility of SMI for CTS diagnosis. We hypothesized that SMI can noninvasively assess blood flow in the median nerve and detect blood flow changes in CTS patients.

## Materials and Methods

### Study Design

**Figure 1:**
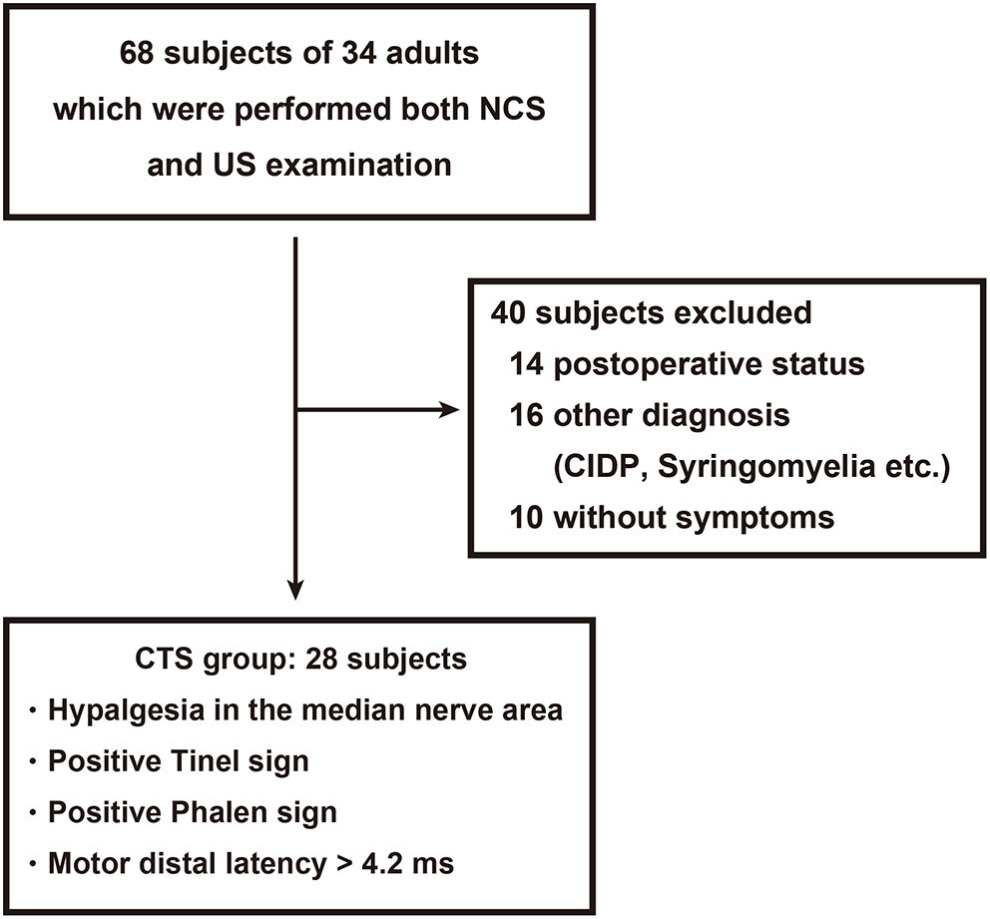
Patient flow diagram. A total of 28 hands from 21 patients with carpal tunnel syndrome (CTS) were enrolled from 68 hands that received both a nerve conduction study and ultrasonography.

This study was approved by our institutional review board (#019-0459 and #020-0304). We performed a retrospective analysis of patients with suspected unilateral or bilateral CTS in our institution from May 2017 to March 2020. Sixty-eight hands from 34 patients who received both NCS and US were enrolled in this study (Figure 1). The inclusion criteria for hands in the CTS group were hypalgesia in the median nerve territory, a positive Tinel sign, a positive Phalen sign, and prolonged motor distal latency (> 4.2 ms) (20). Hands with recurrent CTS, postoperative status, or other neurologic disorders including chronic inflammatory demyelinating polyneuropathy (CIDP) or syringomyelia were excluded. To assess the utility of each method for diagnosing CTS, regardless of the underlying etiology, patients with an orthopedic condition (including fractures), rheumatologic condition, hypertension, diabetes, cardiovascular disease, or other vascular condition were not excluded, and neither were those using tobacco. The control group included the dominant hands from 10 healthy volunteers with no symptoms in the area innervated by the median nerve. The non-dominant hands were also assessed to clarify the influence of laterality in the blood flow analysis. Informed consent was obtained only from healthy volunteers.

### Electrodiagnostic Testing

Studies were performed with a Neuropack MEB-2312 device (Nihon Kohden Corp., Tokyo, Japan) in CTS patients alone, as described previously (21). Standardized skin temperatures and room humidity were maintained during the procedure. In the motor NCS, the active electrode was placed over the muscle berry of the abductor pollicis brevis and the reference electrode was placed just distal to the first metacarpophalangeal joint. The median nerves were stimulated supramaximally at a distance of 6 cm proximal to the recording electrode, and the latency and amplitude of the compound muscle action potential (CMAP) were recorded. No patient had a Martin-Gruber anastomosis.

### Ultrasonography

**Figure 2:**
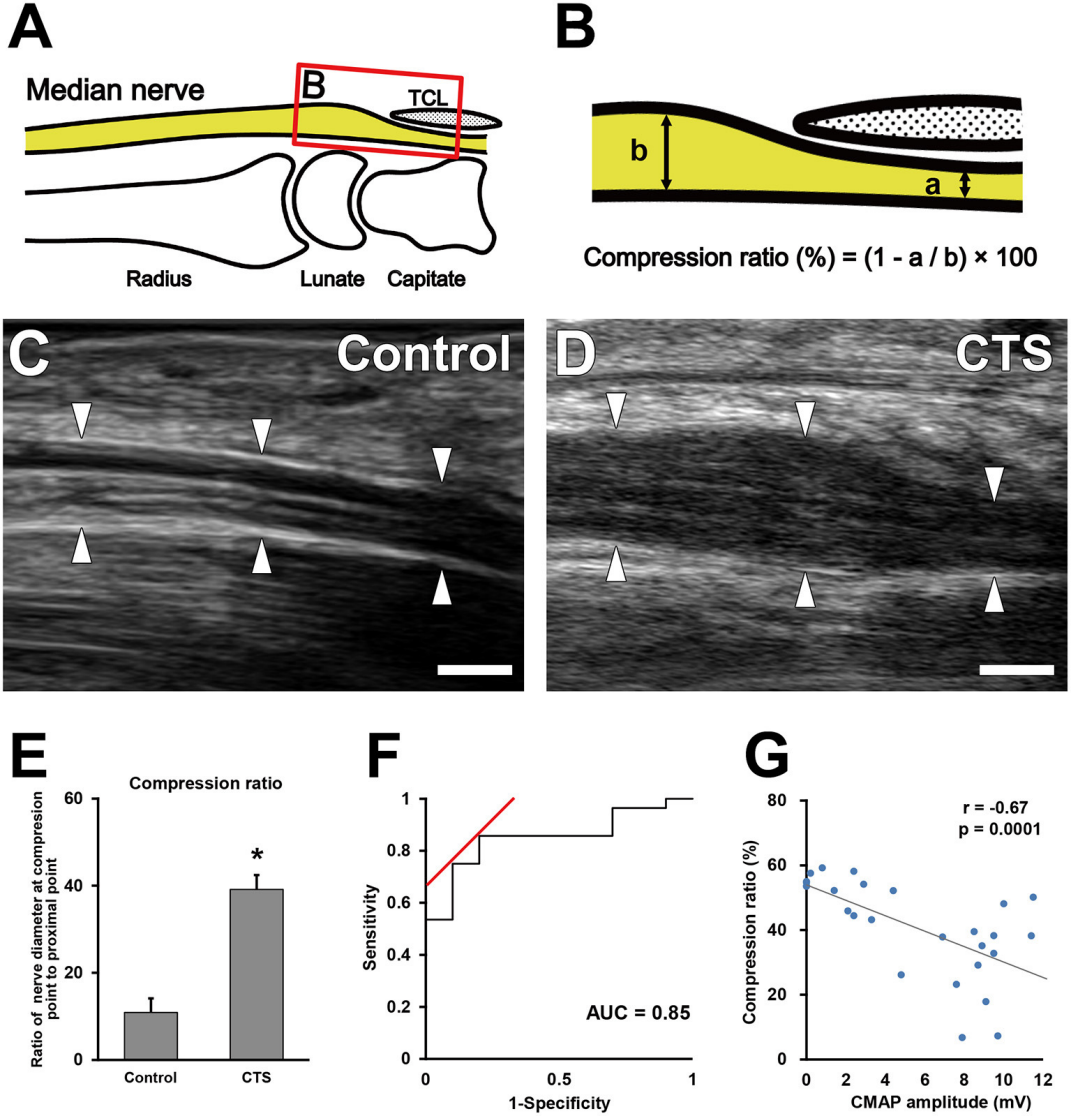
Morphological analysis of the median nerve around the carpal tunnel. Illustration indicates the observed area on ultrasonography **(A)**. **(B)** Boxed area in **(A)**. Quantification of the minimum diameter under the transverse carpal ligament (a) and the maximum diameter around the inlet of the carpal tunnel (b). Ultrasonography of the median nerve in the control **(C)** and carpal tunnel syndrome (CTS) **(D)** groups. Arrowheads indicate the outline of the median nerves. Scale bars: 2 mm. Quantification of the nerve compression ratio **(E)**. *P < 0.05, Student's *t*-test. Data are presented as mean ± standard error of the mean. Receiver operating characteristic curve for the median nerve compression ratio **(F)**. Correlation between the extent of nerve compression and electrophysiological severity **(G)**. Gray line indicates the regression line. The Pearson correlation coefficient = −0.67.

**Figure 3:**
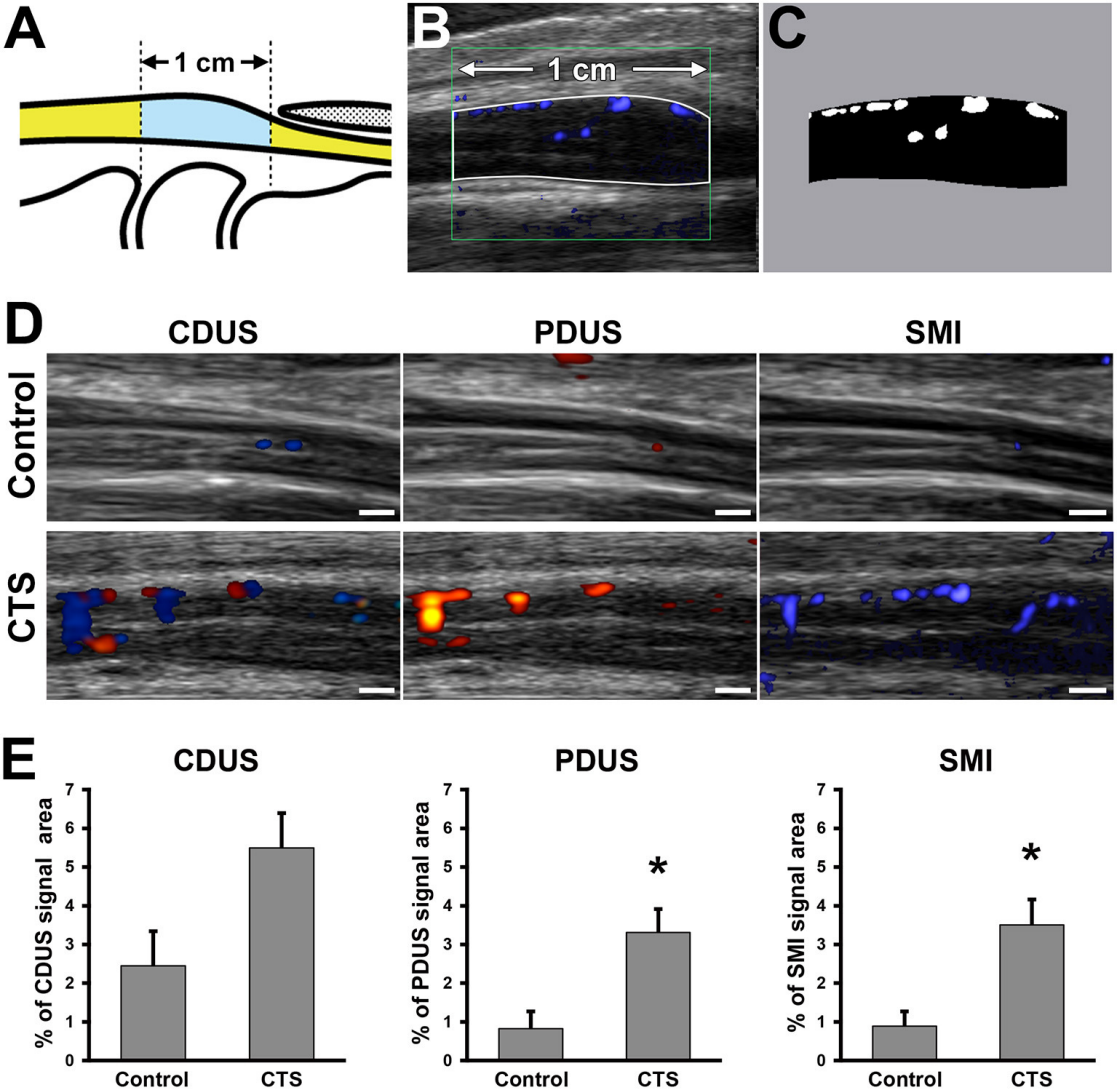
Blood flow signal analysis of the median nerve around the carpal tunnel. Illustration indicates the observed area on ultrasound examination **(A)**. Representative ultrasonography image **(B)**. White line indicates the 1-cm wide analyzed region. **(C)** Bifurcated image of the analyzed region in **(B)**. White pixels indicate the blood flow signal area. Black pixels indicate other areas. Ultrasonography of the median nerve using color Doppler ultrasound (CDUS), power Doppler US (PDUS), and superb microvascular imaging (SMI) **(D)**. Scale bars: 1 mm. Quantification of the blood flow signal area with each technique **(E)**. *P < 0.05, Student's *t*-test. Data are presented as mean ± standard error of the mean.

US (Aplio i800 equipped with a PLI-2004BX transducer [8–24 MHz]; Canon Medical Systems, Otawara, Tochigi, Japan) was performed in the sitting position in all patients, with their wrists in the neutral position. Room temperature was ~24–27°C. All ultrasound examinations were performed by experienced sonographers who were blinded to the findings of physical examinations and the nerve conduction study. On the basis of US findings, morphologic and blood flow analyses were performed using a sagittal section of the median nerve (Figure 2A). For the morphologic analysis, the nerve compression ratio was quantified by calculating the minimum diameter under the transverse carpal ligament (Figure 2Ba) and the maximum diameter around the inlet of the carpal tunnel (Figure 2Bb). The compression ratio (%) was defined as (1–a/b) × 100. Blood flow analysis was performed using CDUS, PDUS, and SMI. The frame rates of CDUS, PDUS and SMI ranged from 11 to 19, 17–23, and 28–59 frame/s, and the velocity ranges were 3.1, 3.1, and 1.2 cm/s, respectively. The frequency was 12 MHz in all groups, and the gain was set to the maximum value of the discrepancy in which the noise disappeared. The blood flow signal area was measured with ImageJ software (v1.51r; https://imagej.nih.gov/ij/index.html) (22) in a 1-cm wide region between the distal edge of the radius and the proximal edge of the transverse ligament (Figures 3A,B). To uniformly count the colored pixels of the blood flow signal area, the “Color Threshold” command was used with threshold values of 64 for saturation and brightness. The blood flow signal area (%) was defined as the total number of pixels of the blood signal area/the total number of pixels of the quantified area (Figure 3C).

### Statistical Analysis

The normality of data distributions was assessed with the Shapiro–Wilk test. Two-group comparisons between the CTS and control groups were performed with the unpaired two-tailed Student's *t*-test. Two-group comparisons between dominant and non-dominant hands were performed with the paired two-tailed Student's *t*-test. The receiver operating characteristic (ROC) curve was used to determine the cutoff value for the compression ratio and the blood flow signal area between the CTS and control groups. Correlation analyses between the blood flow signal area, the distal latency, and the nerve compression ratio were also performed. Interobserver reproducibility of measurement of blood flow signal area were calculated on the basis of measurements performed by three operators on the same subject. Interclass coefficient values of ≥0.80 were considered to indicate acceptable reproducibility. Data are presented as mean ± standard error of the mean. All statistical analyses were performed using statistical software (JMP; SAS, Cary, NC, USA) with a prespecified significance level of 95%.

## Results

A total of 28 hands from 21 patients were enrolled in the CTS group. Between the CTS group and the control group, there were no significant differences in age (62.9 ± 14.1, 56.0 ± 4.4, respectively) or sex (Male:Female, 8:20, 3:7, respectively). The control group included a patient with a history of ipsilateral distal radius fracture. The CTS group included a patient with a history of ipsilateral distal radius fracture, a patient with rheumatoid arthritis, and two patients with hypertension. In the CTS group, the mean distal latency and amplitude of the CMAP were 6.4 ± 1.8 ms (absent in 4 hands) and 5.8 ± 4.0 mV, respectively.

### Nerve Compression Ratio

The nerve compression ratio was significantly higher in the CTS group compared with the control group (40.1 ± 15.1% vs. 13.1 ± 11.9%, respectively; Figures 2C–E). The cutoff value of the nerve compression ratio was 23%, with a sensitivity of 85%, a specificity of 80%, and an area under the ROC curve of 0.85 (Figure 2F). The nerve compression ratio was significantly correlated with the CMAP amplitude (Figure 2G; *r* = −0.67, *P* = 0.0001).

### Blood Flow Signal Area

**Figure 4:**
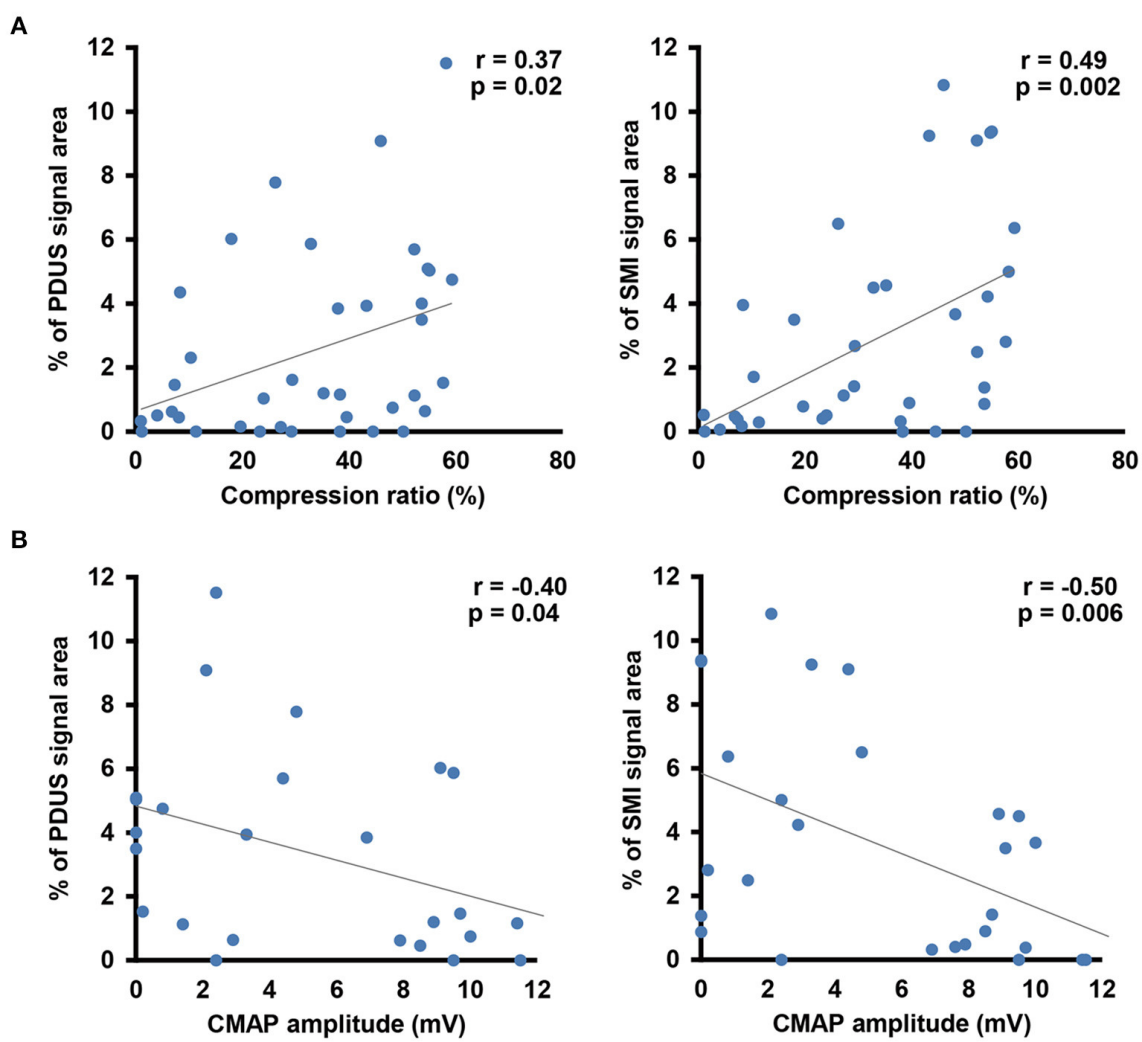
Correlation analysis between the blood flow signal and morphological changes in the median nerve **(A)** or the electrophysiological severity of median nerve neuropathy **(B)**. Gray line indicates the regression line.

In the control group, there were no differences in the blood flow signal area between dominant and non-dominant hands (Supplementary Figure 1). The interclass correlation coefficient for interobserver reliability in blood flow analysis was 0.978 on CDUS, 0.995 on PDUS, and0.995 on SMI. Although there were no differences in the blood flow signal area using CDUS, both PDUS (3.3 ± 0.6% vs. 0.8 ± 0.5%, respectively) and SMI (3.5 ± 0.7% vs. 0.9 ± 0.4%, respectively) showed a significantly higher blood flow signal in the CTS group compared with the control group (Figure 3D). Interestingly, the blood flow signal area detected using SMI had a stronger correlation with the nerve compression ratio (*r* = 0.49) and CMAP amplitude (*r* = −0.50) than for PDUS (*r* = 0.37 and *r* = −0.40, respectively) (Figures 4A,B).

## Discussion

There is accumulating evidence that US is useful for CTS diagnosis. The utility of CSA measurement of the median nerve is well-established (7, 20, 23–26). However, a wide range of recommended CSA cutoff values have been reported (6.5–12.3 mm^2^). In the present study, we used the sagittal view of the median nerve, which enables simultaneous assessment of the median nerve swelling point and the region of median nerve compression, to quantify the median nerve compression ratio. We found that a threshold value of 23% provided a sensitivity and specificity of 85 and 80%, respectively. Furthermore, there was a strong correlation between the compression ratio and the severity of neuropathy in NCS. Overall, these findings support the utility of morphological assessment in the sagittal plane for assisting CTS diagnosis as well as CSA measurement.

We also found that the PDUS and SMI techniques detected blood flow signal changes in the median nerve in CTS patients. Increased blood flow in the median nerve was previously reported in CTS patients using contrast-enhanced US (17), although this technique is not widely used because of its cost and invasiveness. By contrast, CDUS, PDUS, and SMI are non-invasive techniques for visualizing blood flow signals in soft tissues, with previous reports showed their utility in detecting increased median nerve blood flow in CTS patients (10, 18, 27, 28). The SMI technique, which enables visualization of low volume and low velocity blood flow signals, was reported as superior to CDUS and PDUS (18, 29). However, those reports had several limitations. First, they used semiquantitative grading systems involving manual counting of color-encoded spots or lines, without assessment of interobserver and intraobserver reliability. Second, their data showed a ceiling effect because vascularity was classified into only 4 groups based on the number and morphology of the blood flow signal areas, with no separate classifications for blood flow signal counts ≥3. As such, the utility of SMI for assessing CTS remains controversial and further meta-analyses are required (19). Additionally, those studies only used the Aplio500 device. Thus, to our knowledge there are currently no reports of SMI using the latest Aplio i800 model.

A strength of the present study was that we used a uniform threshold value to quantify the color-encoded area. We found that both PDUS and SMI detected an increased blood flow signal in the CTS group, while CDUS did not. This limitation of CDUS may relate to artifacts such as the blooming effect (30, 31), which can cause the relatively higher blood flow signal area in the control group compared with PDUS and SMI. We also found that the blood flow signal area measured using SMI had a relatively stronger correlation with the median nerve compression ratio and neuropathy severity (measured by NCS) compared with PDUS. Overall, these findings suggest that SMI, which enables visualization of low volume and low velocity blood flow (18, 19, 29), is better than CDUS and PDUS for assessing median nerve pathology in CTS patients.

In the present study, we found a significant correlation between increased blood flow and neuropathy severity in CTS patients, although the correlation coefficient was not high. This may be explained, at least in part, by the variance in blood flow changes in CTS patients, especially in electrophysiologically severe cases. The underlying mechanism of neuropathy may differ between CTS patients with or without increased blood flow. Thus, further studies are required to determine the mechanisms of blood flow changes in CTS, for which SMI is particularly useful.

This study has several limitations. First, the analysis was retrospective and based on a small number of patients. Second, blood flow analysis was based on 2-dimensional US images, which may not reflect the entire intraneural blood flow signal. Third, we only assessed patients' hands that had a prolonged distal latency in NCS, while CTS patients with a normal NCS (32) were not included. Lastly, we did not examine the relationship between symptom severity and the blood flow signal level, which would provide further information on the influence of blood flow changes on neuropathy in CTS patients.

In summary, morphological analysis of the median nerve and blood flow analysis using the PDUS and SMI techniques were useful for non-invasive diagnosis of CTS. In particular, blood flow changes detected by SMI showed the highest correlation with the nerve compression ratio and the severity of neuropathy in NCS.

## Data Availability Statement

The datasets generated for this study are available on request to the corresponding author.

## Ethics Statement

The studies involving human participants were reviewed and approved by Institutional Review Board of Hokkaido University Hospital for Clinical Research Hokkaido University Hospital. Written informed consent for participation was required only for healthy volunteers in accordance with the national legislation and the institutional requirements.

## Author Contributions

TE, YM, and NI designed research. TI and MN performed ultrasound examination. DK, AU, DM, and MO contributed unpublished analytic tools. TE analyzed data. YM and HS contributed to analyze interobserver reproducibility. TE and YM wrote the paper. All authors contributed to the article and approved the submitted version.

## Conflict of Interest

The authors declare that the research was conducted in the absence of any commercial or financial relationships that could be construed as a potential conflict of interest.

## Publisher's Note

All claims expressed in this article are solely those of the authors and do not necessarily represent those of their affiliated organizations, or those of the publisher, the editors and the reviewers. Any product that may be evaluated in this article, or claim that may be made by its manufacturer, is not guaranteed or endorsed by the publisher.

## Supplementary Material

Supplementary Figure 1 Blood flow signal analysis of the median nerve around the carpal tunnel in dominant and non-dominant hand of healthy control.
