# Supplementary figures and images for: Diagnostic Utility of Superb Microvascular Imaging and Power Doppler Ultrasonography for Visualizing Enriched Microvascular Flow in Patients With Carpal Tunnel Syndrome
Source: Front Neurol. 2022 Mar 31;13:832569. doi: 10.3389/fneur.2022.832569 (PMC9008197; doi:10.3389/fneur.2022.832569)

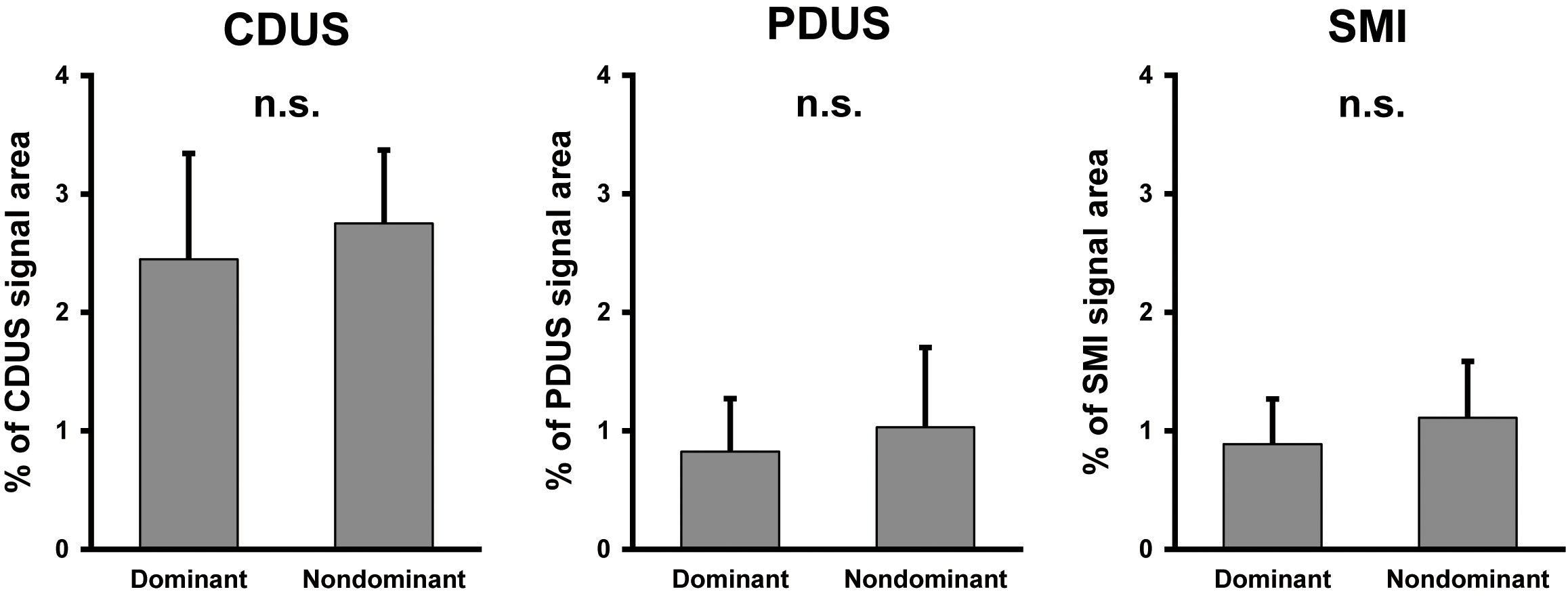

Supplement: Supplementary Figure 1 — Blood flow signal analysis of the median nerve around the carpal tunnel in dominant and non-dominant hand of healthy control. [file Image_1.TIF]
